# Impact of long-term lifestyle programmes on weight loss and cardiovascular risk factors in overweight/obese participants: a systematic review and network meta-analysis

**DOI:** 10.1186/2046-4053-3-130

**Published:** 2014-10-30

**Authors:** Lukas Schwingshackl, Sofia Dias, Georg Hoffmann

**Affiliations:** 1 Faculty of Life Sciences, Department of Nutritional Sciences, University of Vienna, Althanstraße 14 UZA II, A-1090 Vienna, Austria; 2 School of Social & Community Medicine, University of Bristol, Canynge Hall, 39 Whatley Road, Bristol BS8 2PS, UK

**Keywords:** Lifestyle, Obesity, Network meta-analysis, Systematic review, Diet, Exercise

## Abstract

**Background:**

The aim of this meta-analysis was to compare the long-term efficacy of diet plus exercise (D + E) vs. diet (D), D + E vs. exercise (E) and D vs. E on anthropometric outcomes and cardiovascular risk factors in overweight and obese participants.

**Methods:**

Electronic searches were performed in MEDLINE and the Cochrane Central Register of controlled trials. Inclusion criteria were as follows: body mass index ≥25 kg/m^2^ and a minimum intervention period including follow-up of ≥12 months. Outcomes of interest were as follows: anthropometric parameters, blood lipids, blood pressure and cardiorespiratory fitness. Pooled effects were calculated using pairwise random effects and Bayesian random effects network meta-analysis. Results of the corresponding fixed effects models were compared in sensitivity analyses.

**Results:**

Overall, 22 trials (24 reports) met the inclusion criteria and 21 (including 3,521 participants) of them were included in the quantitative analysis. As compared with D, D + E resulted in a significantly more pronounced reduction in body weight [mean differences (MD): -1.38 kg, 95% confidence interval (CI) -1.98 to -0.79], and fat mass (MD: -1.65 kg, 95% CI -2.81 to -0.49], respectively. When comparing D + E with E, MD in change of body weight (-4.13 kg, 95% CI -5.62 to -2.64), waist circumference (-3.00 cm, 95% CI -5.81 to -0.20), and fat mass (-3.60 kg, 95% CI -6.15 to -1.05) was in favour of combined diet and exercise, respectively. Comparing E vs. D, diet resulted in a significantly more pronounced decrease in body weight (MD: -2.93 kg, 95% CI -4.18 to -1.68), and fat mass (MD: -2.20 kg, 95% CI -3.75 to -0.66). D + E yielded also the greatest reductions with respect to blood lipids and blood pressure when compared to single applications of D and E, respectively. Results from the network meta-analyses confirmed these findings.

**Conclusions:**

Moderate-quality evidence from the present network meta-analysis suggests that D + E can be highly recommended for long-term obesity management. Furthermore, the evidence suggests a moderate superiority of D over E with respect to anthropometric outcomes.

**Systematic review registration:**

PROSPERO CRD42013003906

## Background

In 2008, an estimated 1.4 billion adults were overweight meaning that the prevalence of obesity has more than doubled since 1980. Of these, over 200 million men and nearly 300 million women were obese [1]. Overweight (body mass index (BMI): ≥25 kg/m^2^) and obesity (BMI: ≥30 kg/m^2^) are independent risk factors for non-communicable diseases, especially cardiovascular diseases (CVD) and several types of cancer [2,3]. Exercise and diet are cornerstones in the prevention and management of overweight and obesity. Reductions of fat mass, primarily visceral adipose tissue, are major objectives. Energy expenditure increases with physical activity, especially with aerobic exercise or combined aerobic and resistance training [4,5]. Caloric restriction induces weight loss by negative energy balance. Evidence from meta-analyses indicates that low-carbohydrate diets have slightly more favourable effects on body weight as compared to low-fat diets. Nevertheless, independent of macronutrient composition, the long-term health effects of diets are as yet unknown, and the observed outcomes appear of little clinical significance [6-9]. Regarding exercise training, results from recent network meta-analyses indicate that combined aerobic and resistance exercise is the most effective training modality in the treatment/prevention of overweight/obesity and type 2 diabetes mellitus [4,10].

Previous meta-analyses by Shaw et al. [11] (including trials: ≥3 months length) as well as Wu et al. [12] (≥6 months) focused on intervention trials comparing diet plus exercise (D + E) vs. diet (D) on body weight and BMI as outcome parameters, but anthropometric outcomes such as waist circumference, fat mass, waist to hip ratio and cardiovascular risk factors (blood lipids, blood pressure and cardiorespiratory fitness) were not included. To the best of our knowledge, to date, no meta-analysis has compared the head-to-head and indirect long-term (≥12 months) effects of D + E vs. D vs. E on anthropometric parameters and cardiovascular risk factors. Therefore, the aim of this study was to conduct a systematic review with pairwise and network meta-analysis of randomized controlled trials to combine the direct and indirect evidence on the efficacy of different lifestyle long-term weight-reducing interventions on anthropometric parameters, blood lipids, blood pressure and cardiorespiratory fitness in participants with a BMI ≥25 kg/m^2^.

## Methods

The review protocol has been registered in PROSPERO International Prospective Register of Systematic Reviews (crd.york.ac.uk/prospero/index.asp Identifier: CRD42013003906).

### Literature search

Queries of literature were performed using the electronic databases MEDLINE (between 1966 and June 2014) and the Cochrane Trial Register (until June 2014) with no restrictions to language and calendar date using the following search terms: (*“lifestyle”* OR *“exercise”* OR *“diet”*) AND (*“body weight”* OR *“lipids”*) AND (*“randomized controlled trial”* OR *“randomized”* OR *“clinical trials as topic”* OR *“placebo”* OR *“randomly”* OR *“trial”*) NOT (*“animals”* NOT *“humans”*)*.* Moreover, the reference lists from retrieved articles and systematic reviews and meta-analyses were checked to search for further relevant studies. This systematic review was planned, conducted and reported in adherence to standards of quality for reporting meta-analyses [13]. Literature search was conducted independently by two authors (LS, GH), with disagreements resolved by consensus.

### Eligibility criteria

Studies were included in the meta-analysis if they met all of the following criteria: *(i)* randomized controlled design; *(ii)* minimum intervention period including follow-up of 12 months; *(iii)* body mass index: ≥25 kg/m^2^; *(iv)* comparing D + E vs. D or/and D + E vs. E or/and D vs. E; *(v)* assessment of “primary outcome” markers: body weight (BW), waist circumference (WC), waist-to-hip ratio (WHR), fat mass (FM) and “secondary outcome” markers: total cholesterol (TC), low-density lipoprotein cholesterol (LDL-C), high-density lipoprotein cholesterol (HDL-C), triacylglycerols (TG), diastolic blood pressure (DBP), systolic blood pressure (SBP) and cardiorespiratory fitness (VO_2_ max); *(vi)* participants with coronary heart disease were excluded; *(vii)* report post-intervention mean values (if not available change-from-baseline value scores were used) with standard deviation (or basic data to calculate these parameters: standard error or 95% confidence interval (CI)) according to the Cochrane Handbook [14]; and *(viii)* ≥19 years of age.

### Risk of bias assessment

**Figure 1:**
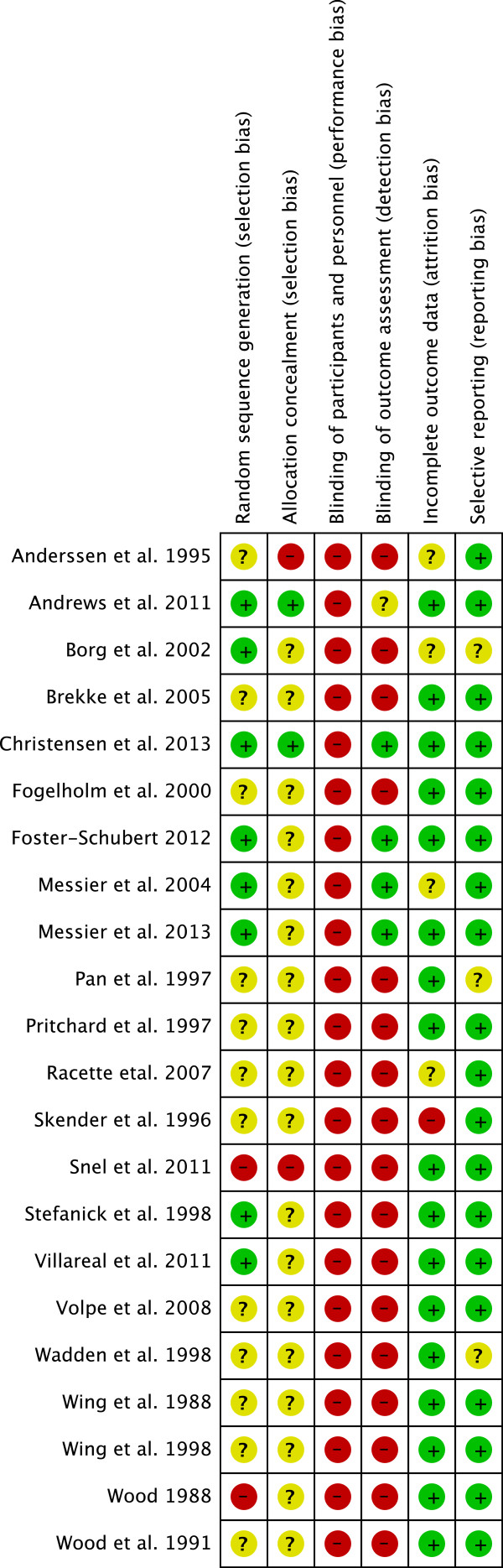
**Risk of bias assessment tool.** Across trials, information is either from trials at a low risk of bias (green), or from trials at unclear risk of bias (yellow), or from trials at high risk of bias (red).

Full copies of studies were independently assessed for methodological quality by two authors (LS, GH) using the risk of bias assessment tool by the Cochrane Collaboration. The following sources of bias were detected: selection bias (random sequence generation, allocation concealment), performance/detection bias (blinding of participants and personnel, blinding of outcome assessment), attrition bias (incomplete data outcome) and reporting bias (selective reporting) (Figure 1) [14,15].

### Data extraction and statistical analysis

The following data were extracted from each study: the first author’s last name, publication year, study length (including follow-up), participant’s sex and age, BMI, sample size,% T2D, intervention type, characteristics of dietary intervention, characteristics of exercise intervention, dropout rates, post-intervention mean values or change-from-baseline value scores with corresponding standard deviation. Data extraction was performed by one author (LS).

Separate pairwise meta-analyses were first used to compare all lifestyle interventions. Network meta-analysis was then used to synthesize all the available evidence [16]. Network meta-analysis methods are extensions of the standard pairwise meta-analysis model which enable simultaneous comparison of multiple interventions whilst preserving the internal randomization of individual trials. They have the advantage of adequately accounting for the correlation in relative effect estimates from three-arm trials as well as providing a single coherent summary of all the evidence.

#### *Pairwise meta-analyses*

For each outcome measure of interest and for each pair of treatments, a random effects inverse variance meta-analysis was performed in order to determine the pooled effect of the intervention in terms of mean differences (MDs) between the post-intervention (or change-from-baseline) values of the different lifestyle interventions [14]. Data were pooled if outcomes were reported by at least three studies. Heterogeneity between trial results was tested with a standard *χ*^2^ test. The *I*^2^ parameter was used to quantify any heterogeneity: *I*^2^ = [(*Q* - *d.f.*)]/*Q* × 100%, where *Q* is the *χ*^2^ statistic and *d.f.* is its degrees of freedom. A value for *I*^2^ > 50% was considered to represent substantial heterogeneity [17]. As study characteristics were expected to differ, statistical heterogeneity was also expected and a random effects model was used to estimate MDs with 95% CIs. In addition, sensitivity analyses were planned to further elucidate the potential influence of heterogeneity due to different study characteristics on the outcome of the pairwise meta-analysis (such as study length, age of participants, risk of bias). Forest plots were generated to illustrate the study-specific effect sizes along with a 95% CI. To determine the presence of publication bias, we assessed the symmetry of the funnel plots in which mean differences were plotted against their corresponding standard errors taking into account the recommendation by Sterne et al. [18], i.e. that testing for funnel plot asymmetry should only be conducted if the number of studies is ten or larger. Additionally, Begg’s and Egger’s regression tests were performed to detect small study effects [19,20].

#### *Network meta-analyses*

To account for the expected between-study heterogeneity, random effects network meta-analysis models were used. For each outcome, a common between-study heterogeneity parameter was assumed to reflect the variability between studies of all interventions.

Model fit was assessed by comparing the number of data points to the posterior mean of the residual deviance [16] (these values should be similar in a well-fitting model). Pooled effect sizes from the network meta-analyses are presented as posterior medians and 95% credible intervals (CrI) (i.e. Bayesian equivalent of confidence intervals) in the appropriate units along with the estimated between-study heterogeneity and its 95% CrI. Treatments were ranked best, second best and third best based on their efficacy.

To assess sensitivity to the choice of random or fixed effects network meta-analysis models, the two models were compared using the deviance information criteria for each outcome [16,21] which account for both model fit and complexity. The fixed effects model was considered adequate when its deviance information criterion (DIC) was lower than the random effects model (differences >3 or 5 are considered meaningful) [16,21]. Mean differences for the fixed effects network meta-analysis (NMA) model are also presented for comparison.

#### *Computation*

For pairwise meta-analyses, data were analysed using the Review Manager 5.1 software, provided by the Cochrane Collaboration (http://ims.cochrane.org/revman). Network meta-analyses were conducted using Markov chain Monte Carlo (MCMC) simulation implemented with the open-source software WinBUGS, version 1.4.3 [22]. The WinBUGS code used is freely available online (program “TSD2-5aRE_Normal_id.odc” for the random effects models and “TSD2-5aFE_Normal_id.odc” for the fixed effects models) [16,23]. Minimally informative normal priors (with mean zero and variance 10,000) were used for all treatment effect parameters, and a uniform (0, 150) prior was used for the between-study standard deviation (heterogeneity) parameter. These priors were considered non-informative over the expected range of data. Sensitivity to the prior on the between-study heterogeneity was assessed by varying the upper bound of the uniform distribution, but there was no meaningful change in relative effects or overall conclusions.

Three MCMC chains were used to assess convergence using Brooks-Gelman-Rubin plots and by inspection of the trace plots [24]. Convergence was achieved after 20,000 iterations for all outcomes. Posterior summaries were then obtained from further simulation of 50,000 iterations in each of the three chains (150,000 in total), resulting in a small Monte Carlo error.

Treatments were ranked at each iteration (post-convergence) according to their efficacy, where the “best” treatment was the one with the most favourable outcome (which could be described by a higher or lower MD, depending on the outcome).

The potential for inconsistency was assessed by inspection of the network plots. Where there was a potential for inconsistency, i.e. where there were independent sources of evidence informing direct and indirect estimates, Bayesian *p* values for the difference between direct and indirect evidence were calculated using the node split method [25,26] implemented in R through GeMTC [27], and direct and indirect estimates were compared.

## Results

**Figure 2:**
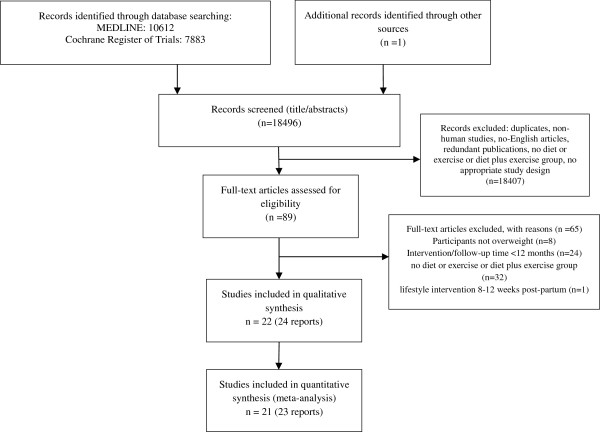
Flow diagram.

In order to ease interpretation of results, the following changes would be considered to be a benefit: BW, decrease; WC, decrease; FM, decrease; WHR, decrease; TC, decrease; LDL-C, decrease; HDL-C, increase; TG, decrease; DBP, decrease; SBP, decrease; VO_2_ max, increase; altogether, 22 trials (24 reports) met the inclusion criteria and 21 of them were included in the quantitative analysis [28-52]. The detailed steps of the meta-analysis article selection process are given as a flow chart in Figure 2, and full search strategy for PUBMED and the Cochrane Trial Register is given in Additional file 1.

All studies included were randomized controlled trials (RCTs) with a duration ranging between 12 and 72 months, published between 1988 and 2013 and enrolling a total of 3,521 participants, 680 of them being participants with T2D. The mean age varied between 35 and 70 years and the BMI between 25.6 and 38.2 kg/m^2^. Seventeen trials compared D + E vs. D, 11 compared D + E vs. E and 14 compared D vs. E. General study characteristics are summarized in Table 1. Regarding the dietary interventions, a major part of the included trials recommended energy-reduced low-fat diets (≤30% fat of total energy), low in saturated fat, and increased intakes of fruit, vegetables and fibre. Exercise prescription was partly supervised and included aerobic exercise (i.e. jogging, walking, flexibility, circuit training) and resistance training, overall 50%–85% of maximal heart rate.

**Table 1:** General study characteristics

**Reference**	**Sample size**	**Age (years)**	**Duration of the active intervention (follow-up)**	**Study design**	**Dietary intervention**	**Dropout**
	**Baseline BMI (kg/m**^**2**^**)**	**Female (%)**			**Exercise prescription**	
	**% diabetics**					
Anderssen et al. [40] Reseland et al. [41]	166	40	12 months	D + E vs. D vs. E	D: increased fish, fruit and vegetables and fibre, reduce intake of sugar and SFA, no heavy evening meal	D + E: 3% D: 5%
	28.9	0%	(0 months)			
						E: 9%
					E: supervised weekly, aerobic training (strength, flexibility, circuit training, jogging), 60%–80% of peak heart rate	
	0%					

Andrews et al. [42]	494	60	12 months	D + E vs. D	D: aimed at enabling patients to lose 5%–10% of their initial body weight, based on UK dietary guidelines E: asked to do at least 30-min brisk walking on at least 5 days per week	D + E: 2% D: 1%
	31.55	35%	(0 months)			
	100%					
Borg et al. [43]	82	42.6	8 months	D + E vs. D	D: low-fat diet	D + E: 18% D: 1%
	32.9	0%	(23 months)		E: supervised weekly in group, reached 50%–60% of MHR, included two groups: (1) walking, expended 1,000 kcal per week, and (2) walking, expended 2,000 kcal per week	
	n.d.					
Brekke et al. [44]	49	43	12 months	D + E vs. D	D: Nordic Nutrition Recommendation in addition increase of low GI food	D + E: 17%
	25.6	33%	(12 months)			
					E: the goal to increase physical activity through walking or other more intensive activities for at least 30 min, four to five times per week	D: 4%
	100%					
Christensen et al. [52]	28	63	17 months	D vs. E	D: the goal of the dietary intervention was to produce and maintain a weight loss of at least 10%	D: 14%
						E: 19%
					E: exercise intervention consisted of a warm-up phase (10 min), a circuit training phase (45 min) and a cool down/stretching phase four periods of 12 weeks and one period of 4 weeks (total 52 weeks). The aim was to gradually translate the intervention from facility-based exercises to home-based exercises	
	37.05	81.3	(0 months)			
	0%					
Fogelholm et al. [45]	82	35	13 months	D + E vs. D	D: low-fat diet	D + E: 2% D: 3%
	34	100%	(11 months)		E: supervised weekly in group, reached 50%–60% of MHR, included two groups: (1) walking, expended 1,000 kcal per week, and (2) walking, expend 2,000 kcal per week	
	0%					
Foster-Schubert et al. [46]	351	58	12 months	D + E vs. D vs. E	D: total daily energy intake 1,200–2,000 kcal/day on baseline weight <30% fat and 10% reduction in body weight by 6 months with maintenance thereafter for 12 months	D + E: 8% D: 11%
						E: 9%
					E: ≥45 min of moderate-to-vigorous intensity exercise, 5 days/week (225 min/week) for 12 months; supervised; 70%–85% of MHR	
	28.6	100%	(0 months)			
	0%					
Messier et al. [51]	238	68.7	18 months	D + E vs. D vs. E	D: the goal of intervention was to produce and maintain an average weight loss of 5% baseline body weight. The intervention was divided into three phases: intensive, transition and maintenance	D + E: 24%
	34.3	73%	(0 months)			
Nicklas et al. [47]	9%					
						D: 23%
						E: 20%
					E: exercise consisted of an aerobic phase, a resistance training phase, a second aerobic phase and a cooling down phase	
Messier et al. [50]	454	66	18 months	D + E vs. D vs. E	D: diet was based on partial meal replacements, including up to two meal replacement shakes per day; for the third meal, participants followed a weekly menu plan and recipes that were 500 to 750 kcal, low in fat and high in vegetables; initial diet plan provided an energy intake deficit of 800 to 1,000 kcal/day	D + E: 11%
	33.6	72%	(0 months)			
						D: 15%
	13%					E: 11%
					E: exercise was conducted for 1 h on 3 days/week for 18 months; programme consisted of aerobic walking (15 min), strength training (20 min), a second aerobic phase (15 min) and cool down (10 min)	
Pan et al. [48]	397	44.4	72 months	D + E vs. D vs. E	D: caloric intake at 25–30 kcal/kg of BW, increased vegetable intake and reduced intake of sugars, using individual goals	D + E: 8%
	25.6	47%	(0 months)			D: 8%
	0%, 100% IGT					
					E: increased the amount of exercise at least 1 U/day and U/day for those less than 50 years old with no evidence of heart disease or arthritis. The rate of increase and type of exercise depending on age, past exercise pattern and existence for heart problem other than IGT	
						E: 8%
Pritchard et al. [39]	39	44.25	12 months	D vs. E	D: low-fat diet	D: 0%
					E: aerobic exercise; minimum participation was three sessions of 30 min per week. 65%–75% of MHR was recommended to achieve maximum weight loss	
						E: 0%
	29.1	0%	(0 months)			
	0%					
Racette et al. [38]	45	57.2	12 months	D vs. E	D: decrease energy intake by 16% for the initial 3 months and by 20% for the remaining 9 months; macronutrient composition was flexible	D: 4% E: 4%
	37.2	63%	(0 months)			
	0%					
					E: the goal of the E intervention was to induce an energy deficit comparable to the CR intervention by increasing daily energy expenditure through exercise without changing caloric intake. Exercise physiologists and trainers worked with ex-participants individually to establish and monitor their exercise routines	
Skender et al. [37]	61	45	12 months	D + E vs. D vs. E	D^a^: help your heart eating plan; well-balanced, low-cholesterol eating plan	D + E: 50%
	35	48%	(12 months)			
					E: supervised weekly although group brisk walking at a level of felt “vigorous” not “strenuous”, 45 min, 4–5 times/week	D: 65%
	0%					E: 42%
Snel et al. [36]	27	57.5	4 months	D + E vs. D	D: 4 months: 450 kcal/day (consisting of three sachets of Modifast); weight maintenance: 1,800 kcal	D + E: 0% D: 0%
	37	48%	(14 months)			
	100%				E: 4 days training at home for 30 min at 70% of maximum aerobic capacity on a cyclo-ergometer and 1 h in hospital training under the supervision of a physiotherapist	
Stefanick et al. [35]	276	56.9/47.8	12 months	D + E vs. D vs. E	D: NCEP step 2 diet	D + E: 3%
	26.3/27	48%	(0 months)		E: supervised weekly, aerobic exercise =16-km jogging per week	D: 3%
	0%					E: 3%
Villareal et al. [34]	80	70	12 months	D + E vs. D vs. E	D: balance diet with an energy deficit of 500 to 750 kcal. The diet contained 1 g of high-quality protein/kg of BW per day	D + E: 11%
	37	61%	(0 months)			
						D: 15%
	n.d.					E: 12%
					E: three supervised exercise training sessions per week. Each session was 90 min in duration and consisted of aerobic and resistance exercise and exercise to improve flexibility and balance	
Volpe et al. [33]	90	44.4	6 months	D + E vs. D vs. E	D: intensive (weekly) nutritional classes (1–3 months)	D + E: n.d.
					E: supervised training on Nordic Track™ indoor skiing apparatus, 3–4 days per week, 30 min for 6 weeks	
	30.5/35.3	51%	(6 months)			
	0%					D: n.d.
						E: n.d.
Wadden et al. [32]	77	42	12 months	D + E vs. E	D: conventional diet with 1,200–1,500 kcal/day	D + E: 22%
	36.5	100%	(0 months)		E: supervised weekly in a group, 1 h, two times per week, included in three exercise groups: (1) aerobic, (2) strength and (3) combined training	E: 22%
	0%					
Wing et al. [29]	114	45.5	24 months	D + E vs. D vs. E	D: participants were asked to follow an 800–100 kcal/day diet, with 20% of calories as fat, exactly as prescribed for 1–8 weeks of the programme. Gradually more flexible with calorie goals adjusted to 1,200–1,500 kcal/day at week 16. Subject attended weekly group meetings for the first 6 months	D + E: 20% D: 5% E: 16%
					E: supervised by exercise physiologists weekly in a group. Mainly brisk walking, 3 miles, five times per week, total activity gradually increased to 1,500 kcal per week	
	35.9	79%	(0 months)			
	0%					
Wing et al. [30]	30	55.56	12 months	D + E vs. D	D: daily calorie goal designed to produce approximately 1 kg/week weight loss. Low-fat diet	D + E: 13%
	38.2	70%	(0 months)			
						D: 0%
	100%				E: all participants exercised twice a week as a group and once a week on their own, with each exercise session lasting approximately 1 h	
Wood et al. [49]	152	39.1/40.3	12 months	D + E vs. D	D^b^: NCEP step 1 diet	D + E: 14%
					E: aerobic exercise (brisk walking and jogging) that met 3 days a week, 60%–80% of MHR for 25–45 min per time (by the fourth month of the study)	
	27.9/30.7	48%	(0 months)			
						D: 13%
	0%					
Wood et al. [28]	89	44.1	12 months	D vs. E	D: individual prescription designed to reduce baseline total body fat by one third over a 9-month period	D: 4%
						E: 2%
	n.d	0%	(0 months)		E: supervised exercise programme and individual prescriptions based on estimates of the amount of energy necessary to decrease total body fat progressively by one third over 9 months	
	0%					

*BMI* body mass index, *BW* body weight, *CR* caloric restriction, *D* diet, *E* exercise, *GI* glycaemic index, *IGT* impaired glucose tolerance, *MHR* maximal heart rate, *NCEP* National Cholesterol Education Program, *SFA* saturated fat, *UK* United Kingdom; *n.d.* no data.

^a^Skender et al. [37]: 50% CH, 30% F, 20% P, low cholesterol.

^b^Wood et al. [49]: 55% CH, 30% F, <10% SFA, <300 mg/day.

The direct pairwise and network pooled estimate of effect size for the effects of D + E vs. D, D + E vs. E and D vs. E on anthropometric outcomes, blood lipids, blood pressure and cardiorespiratory fitness are summarized in Table 2.

**Table 2:** **Estimates (direct pairwise and network meta-analysis, random effects models) of effect size (95% confidence intervals/95% credible intervals) expressed as mean difference for the effects of diet + exercise vs. diet, diet + exercise vs. exercise and diet vs. exercise on anthropometric outcomes, blood lipids, blood pressure and cardiorespiratory fitness and between-study heterogeneity variance (*τ***^**2**^**/ *τ *)**

**Outcomes**	**No. of studies**	**Sample size**	**MD**	**95% CI**	***τ***^**2**^	***I***^**2**^	**MD**	**95% CrI**	***τ***	**95% CrI**
**D + E vs. D**										
BW (kg)	17	2,317	-1.38	[-1.98, -0.79]	0.00	0%	-1.38	[-2.62, -0.17]	2.06	[1.37, 2.96]
WC (cm)	8	1,124	-1.68	[-2.66, -0.70]	0.00	0%	-1.69	[-3.32, -0.20]	1.36	[0.07, 3.43]
FM (kg)	9	1,012	-1.65	[-2.81, -0.49]	1.95	61%	-1.89	[-3.44, -0.43]	2.08	[1.29, 3.24]
WHR (U)	6	646	-0.01	[-0.02, -0.01]	0.00	0%	-0.01	[-0.05, 0.03]	0.06	[0.04, 0.10]
TC (mg/dl)	9	1,175	-2.19	[-7.84, 3.46]	54.09	62%	-2.51	[-7.61, 2.29]	5.42	[0.86, 10.84]
LDL-C (mg/dl)	8	1,147	-0.93	[-6.14, 4.27]	45.03	65%	-1.54	[-6.16, 3.14]	5.29	[1.26, 9.97]
HDL-C (mg/dl)	9	1,175	1.62	[0.28, 2.95]	2.46	51%	-1.29	[-1.38, 3.86]	4.03	[2.38, 6.33]
TG (mg/dl)	9	1,175	-10.08	[-17.38, -2.79]	0.00	0%	-9.90	[-19.98, -0.96]	7.28	[0.37, 19.4]
DBP (mmHg)	7	1,099	-1.20	[-2.26, -0.15]	0.74	28%	-1.10	[-2.34, 0.01]	0.92	[0.04, 2.45]
SBP (mmHg)	7	1,099	-0.24	[-1.45, 0.97]	0.00	0%	-0.39	[-1.89, 1.01]	0.87	[0.04, 2.62]
VO_2_ max (ml/kg/min)	6	810	3.61	[2.07, 5.14]	4.19	88%	3.75	[2.28, 5.32]	1.94	[1.13, 3.18]
**D + E vs. E**										
BW (kg)	9	1,350	-4.13	[-5.62, -2.64]	4.36	77%	-4.32	[-5.74, -2.90]	2.06	[1.37, 2.96]
WC (cm)	3	409	-3.00	[-5.81, -0.20]	5.24	69%	-3.45	[-5.32, -1.23]	1.36	[0.07, 3.43]
FM (kg)	5	690	-3.60	[-6.15, -1.05]	8.82	92%	-3.87	[-5.61, -2.18]	2.08	[1.29, 3.24]
WHR (U)	4	420	-0.01	[-0.02, -0.00]	0.00	15%	-0.007	[-0.06, 0.04]	0.06	[0.04, 0.10]
TC (mg/dl)	4	420	-11.36	[-15.93, -6.79]	0.00	0%	-7.50	[-13.47, -1.39]	5.42	[0.86, 10.84]
LDL-C (mg/dl)	4	420	-10.03	[-14.28, -5.78]	2.22	8%	-5.90	[-11.39, -0.23]	5.29	[1.26, 9.97]
HDL-C (mg/dl)	4	420	-0.34	[-2.82, 2.14]	6.80	76%	0.17	[-3.14, 3.32]	4.03	[2.38, 6.33]
TG (mg/dl)	4	420	-11.18	[-26.99, 4.62]	138.3	38%	-13.34	[-25.92, -2.12]	7.28	[0.37, 19.4]
DBP (mmHg)	4	420	-2.06	[-3.39, -0.72]	0.00	0%	-2.22	[-3.93, -0.74]	0.92	[0.04, 2.45]
SBP (mmHg)	4	420	-2.84	[-4.54, -1.13]	0.00	0%	-2.70	[-4.57, -0.85]	0.87	[0.04, 2.62]
VO_2_ max (ml/kg/min)	5	645	2.13	[1.52, 2.74]	0.05	9%	2.24	[0.57, 3.90]	1.94	[1.13, 3.18]
**D vs. E**										
BW (kg)	13	1,638	-2.93	[-4.18, -1.68]	3.70	73%	-2.93	[-4.20, -1.66]	2.06	[1.37, 2.96]
WC (cm)	4	539	-1.75	[-4.12, 0.62]	4.80	71%	-1.76	[-3.48, 0.44]	1.36	[0.07, 3.43]
FM (kg)	9	964	-2.20	[-3.75, -0.66]	4.66	82%	-1.97	[-3.45, -0.45]	2.08	[1.29, 3.24]
WHR (U)	4	414	-0.00	[-0.01, 0.01]	0.00	16%	0.002	[-0.05, 0.05]	0.06	[0.04, 0.10]
TC (mg/dl)	7	665	-3.91	[-8.11, 0.30]	9.04	22%	-4.98	[-10.22, 0.64]	5.42	[0.86, 10.84]
LDL-C (mg/dl)	7	665	-3.19	[-6.85, 0.48]	6.45	21%	-4.36	[-9.25, 0.70]	5.29	[1.26, 9.97]
HDL-C (mg/dl)	7	665	-0.96	[-1.88, -0.04]	0.00	0%	-1.12	[-4.06, -1.76]	4.03	[2.38, 6.33]
TG (mg/dl)	7	665	-3.80	[-12.21, 4.62]	0.00	0%	-3.44	[-13.99, 6.65]	7.28	[0.37, 19.4]
DBP (mmHg)	6	573	-1.33	[-3.00, 0.35]	11.19	37%	-1.12	[-2.67, 0.31]	0.92	[0.04, 2.45]
SBP (mmHg)	6	578	-2.19	[-4.23, -0.15]	2.07	25%	-2.31	[-4.10, 0.51]	0.87	[0.04, 2.62]
VO_2_ max (ml/kg/min)	6	677	-1.16	[-2.42, 0.09]	2.17	80%	-1.15	[-3.16, 0.04]	1.94	[1.13, 3.18]

*BW* body weight, *CI* confidence intervals, *D* diet, *DBP* diastolic blood pressure, *D + E* diet and exercise, *E* exercise, *FM* fat mass, *HDL-C* high-density lipoprotein cholesterol, *LDL-C* low-density lipoprotein cholesterol, *SBP* systolic blood pressure, *TC* total cholesterol, *TG* triacyglycerols, *VO*_*2*_*max* maximal oxygen uptake, *WC* waist circumference, *WHR* waist-to-hip ratio.

### Anthropometric outcomes/cardiorespiratory fitness

#### *Diet + exercise vs. diet*

The weighted mean difference in change of BW [MD: -1.38 kg (95% CI -1.98 to -0.79), *I*^2^ = 0%], WC [MD: -1.68 cm (95% CI -2.66 to -0.70), *I*^2^ = 0%], WHR [MD: -0.01 U (95% CI -0.02 to -0.01), *I*^2^ = 0%] and FM [MD: -1.65 kg (95% CI -2.81 to -0.49), *I*^2^ = 61%] was significantly more pronounced in the D + E group as compared to D, respectively. Furthermore, the D + E group revealed significantly more prominent increases in cardiorespiratory fitness (measured as VO_2_ max) [MD: 3.61 ml/kg/min (95% CI 2.07 to 5.14), *I*^2^ = 88%] and HDL cholesterol [MD: 1.62 mg/dl (95% CI 0.28 to 2.95), *I*^2^ = 51%] as well as decreases in TG [MD: -10.08 mg/dl (95% CI -17.38 to -2.79), *I*^2^ = 0%] and DBP [MD: -1.20 mmHg (95% CI -2.26 to -0.15), *I*^2^ = 28%]. In contrast, changes observed for TC, LDL-C and SBP did not differ significantly between both groups.

#### *Diet + exercise vs. exercise*

Comparing D + E vs. E, a significantly more distinctive reduction in BW [MD: -4.13 kg (95% CI -5.62 to -2.64), *I*^2^ = 77%], WC [MD: -3.00 cm (95% CI -5.81 to -0.20), *I*^2^ = 69%], WHR [MD: -0.01 U (95% CI -0.02 to -0.00), *I*^2^ = 15%] and FM [MD: -3.60 kg (95% CI -6.15 to -1.05), *I*^2^ = 92%] could be observed in the D + E group. Rise of VO_2_ max was significantly more pronounced in the D + E group as well [MD: 2.13 ml/kg/min (95% CI 1.52 to 2.74), *I*^2^ = 9%]. With respect to blood lipids, TC [MD: -11.36 mg/dl (95% CI -15.93 to -6.79), *I*^2^ = 0%], LDL-C [MD: -10.03 mg/dl (95% CI -14.28 to -5.78), *I*^2^ = 8%], DBP [MD: -2.06 mmHg (95% CI -3.39 to -0.72), *I*^2^ = 0%] and SBP [MD: -2.84 mmHg (95% CI -4.54 to -1.13), *I*^2^ = 0%] were reduced more substantially following combined D + E when compared to single E interventions. No significant differences could be observed for HDL-C and TG.

#### *Diet vs. exercise*

Following D vs. E comparisons, reductions in BW [MD: -2.93 kg (95% CI -4.18 to -1.68), *I*^2^ = 74%], FM [MD: -2.20 kg (95% CI -3.75 to -0.66), *I*^2^ = 82%], HDL-C [MD: -0.96 mg/dl (95% CI -1.88 to -0.04), *I*^2^ = 0%] and SBP [MD: -2.19 mmHg (95% CI -4.23 to -0.15), *I*^2^ = 25%] were significantly more pronounced in the D group. WC, WHR, TC, LDL-C and cardiorespiratory fitness were not affected in a different fashion by either D or E.

### Network meta-analysis

Additional file 1: Figure S1 shows the network of included trials. The pooled estimates of effect size for the comparison of D + E vs. D vs. E using both direct and indirect evidence on anthropometric outcomes, blood lipids, blood pressure and cardiorespiratory fitness are summarized in Table 2.

Both D + E and D were significantly more effective in reducing BW and FM when compared to E alone. D + E turned out to be the most effective lifestyle intervention with respect to reduction of BW, WC, FM, TC, LDL-C, TG, DBP, SBP and increasing HDL-C. D + E resulted in a high (>75%) probability to be the best for most outcomes. There is greater uncertainty regarding which treatment is the best for HDL-C, although again D + E yielded the highest probability of being the best. D turned out be the second effective lifestyle intervention for BW, WC, FM, TC and DBP (>75% probability).

There was potential for inconsistency in the networks for all outcomes except WHR (Additional file 1: Figure S1). There was some evidence of inconsistency for the outcome LDC (*p* value = 0.02) although this might be due to chance since several *p* values for inconsistency are being calculated (Additional file 1: Table S8). After inspection of the evidence on this outcome we did not identify a reason for this apparent inconsistency.

### Risk of bias

The dropout rates ranged from 0% to 65%, with 11 out of 22 trials reporting dropout rates <10% (Table 1). Pairwise meta-analysis resulted in no significant dropout differences (*p* = 0.37) between D + E (122/1,257) vs. D (111/1,152), D + E (79/714) vs. D (82/715) (*p* = 0.96) and D + E (87/701) vs. D (84/706) (*p* = 0.34). Eight trials (nine reports) reported random sequence generation [34,35,42,43,46,47,50-52], and only two trials reported allocation concealment [42,52]. None of the studies reported blinding of volunteers towards mode of intervention (not possible in diet/exercise trials), and four trials (five reports) appear to have adequate blinding of outcome assessment [46,47,50-52]. High risk of bias was defined as fewer than three out of a maximum yield of six low risk of bias items using the risk of bias assessment tool from the Cochrane Collaboration. Only seven low risk of bias trials (eight reports) were identified [34,35,42,46,50-52], and sensitivity analyses were performed for studies with a low risk of bias (Additional file 1: Table S1).

### Sensitivity analysis

Sensitivity analyses (pairwise meta-analysis) were performed for obese, study length ≥24 months, older participants (age: ≥50 years) and low risk of bias studies. Comparison of long-term vs. short-term trials resulted in smaller reductions in body weight (Additional file 1: Tables S2-S3). The results of the primary analysis (D + E vs. D and D vs. E) could be confirmed including only obese participants (Additional file 1: Table S4), except for WC and VO_2_ max. Including only participants ≥50 years of age (Additional file 1: Table S5), results of the primary analysis could not be confirmed regarding anthropometric outcomes when comparing D + E vs. D (only four to eight trials available), whilst the results of the main analysis for D vs. E were not affected. Sensitivity analyses excluding trials with a high risk of bias changed some summary estimates (D + E vs. D) for anthropometric outcomes and became statistically non-significant (with the exception of D vs. E comparisons).

#### *Fixed vs. random effects models*

Results of the pairwise meta-analyses with a fixed effects model are presented in Additional file 1: Table S6. Overall, the results of the main analysis could be confirmed in the fixed pairwise sensitivity analyses. However comparing D vs. E resulted in a significant reduction in WC, TC and LDL-C in the fixed effects model, which was not observed in the random effects model, that were more pronounced in the D group. However, we note that the between-study heterogeneity was moderate to high for these outcomes and therefore the fixed effects model may not be appropriate.

In the NMA, comparing the DIC for fixed and random effects models suggests that the fixed effects model might be appropriate for DBP, SBP, TC, TG and WC (Additional file 1: Table S7). The estimated treatment effects using fixed effects network meta-analyses are given in Additional file 1: Table S6. Some differences could be observed between the random and fixed effects models. In the fixed effects model, comparing D vs. E resulted in a significant reduction in WC, TC, LDL-C, SBP and VO_2_ max, favouring D (with the exception of VO_2_ max), compared to the random effects model. Furthermore, the fixed effects model showed a significant reduction in WHR, comparing D + E vs. D/E, and increase in HDL-C compared to E. Again, we note that the fixed effects model was not always considered suitable (Additional file 1: Table S7).

### Publication bias

Begg's and Egger's regression tests provided no evidence of substantial publication bias (data not shown). Funnel plots were generated only if specific outcome measures were provided by at least ten different trials (some trials provide more than one data point for analysis as they report results for subgroups: men and women) [18]. The plot with respect to effect size change for body weight in response to D + E vs. D vs. E indicates little asymmetry (Additional file 1: Figures S2-S3). Thus, publication bias cannot be completely excluded as a factor affecting the results of the present meta-analysis.

## Discussion

The primary findings of this systematic review and meta-analysis of long-term trials are that a combination of D + E is more effective in reducing BW compared to either D or E in overweight and obese participants. Furthermore, improvements in WC, WHR and FM were significantly more pronounced following D + E compared to either D or E. The combination of D + E is also the most powerful lifestyle intervention to reduce blood lipids and blood pressure. Compared to E, a dietary intervention resulted in significantly greater reductions in body weight, fat mass and systolic blood pressure. Pooling both direct and indirect evidence on D + E, D and E via network meta-analysis demonstrated that D + E was the most efficacious exercise intervention regarding its impact on BW, WC, FM, TC, LDL-C, TG, DBP and VO_2_ max.

The findings of this meta-analysis, i.e. diet being significantly more effective in reducing BW than exercise, are supported by results of a 2006 Cochrane review [11] and are consistent with results of another meta-analysis [53]. Therefore, caloric restriction appears the most powerful method for achieving weight loss in overweight and obese people. The amount of weight loss yielded by diet and exercise can be compared to the corresponding results of the most effective pharmacological interventions such as orlistat (4.12/3.1 kg at 12 months) [54,55]. A recent systematic review concluded that orlistat, lorcaserin and phentermine/topiramate ER when used as an adjunct to lifestyle intervention, could induce a clinically relevant (≥5%) 12-month weight loss [56]. The interpretation of our network meta-analysis is restricted by the fact that none of the trials evaluated the impact of their interventions on clinical outcomes. It should be noted, however, that no anti-obesity drugs have been shown to have a favourable effect on CVD morbidity and mortality as well [57]. Furthermore anti-obesity drugs were associated with increased risk of total adverse events, tachycardia, gastrointestinal disease, hypertension and mouth dryness [58].

Previous studies reported that a 5% reduction of BW is associated with a reduced risk of T2D incidence and other metabolic disorders. A 5-kg weight loss over time could account for a 55% reduction in the risk of diabetes over the mean of 3.2 years of follow-up in a high-risk population [59]. Regarding visceral adipose tissue, a comprehensive meta-analysis of cohort studies showed that a 1-cm increase in WC and a 0.01-U increase in WHR are associated with a 2%–5% increase in risk of future CVD [60]. Applying this data to the results of the present meta-analysis, D + E would be associated with a CVD risk reduction of ~3%–6%. Improvements on anthropometric outcomes were more distinct in younger participants compared with older which might be explained by the fact that younger participants were able to perform more intense exercise sessions. However, since not all trials applied supervised exercise, no definitive explanation can be given.

With respect to blood lipids, a meta-analysis of 70 studies indicate that each kilogram of weight loss was associated with a 1.9 mg/dl decrease in TC and a 0.77 mg/dl decrease in LDL-C, respectively [61]. Furthermore, RCTs showed that especially aerobic exercise was associated with an increase in HDL-C [62]. These associations could be confirmed in the present meta-analysis.

The predominant dietary intervention implemented in the included trials was either an energy-restricted low-fat diet or an energy-balanced moderate-fat diet. In general, the composition of diets was approximately at least 500 kcal below the estimated energy need, and fat intake was ≤30% of total energy content. In the D + E and D trials, 1,200 kcal for women and 1,500 kcal for men were generally prescribed. In the dietary intervention trials, general guidelines for physical activity were recommended. However, in the D + E trials, specific goals for physical activity/exercise were implemented. Network meta-analyses provides evidence that a combination of aerobic and resistance training should be recommended in the prevention and treatment of overweight, obesity and associated diseases [4,10].

Cardiorespiratory fitness is associated with cardiovascular mortality and cancer in men and women [63,64]. A pooled analysis investigating the effects of cardiorespiratory fitness on all-cause mortality and cardiovascular events demonstrated that a 1-unit increase in metabolic equivalents was associated with a 13% and 15% reduction in risk of all-cause mortality and coronary heart disease (CHD)/CVD, respectively [65]. Transferring this data to the results of the present meta-analysis, D + E reduced the risk of all-cause mortality by 14% and of CVD/CHD by 16% and approximately 8% (mortality) and 9% (CVD/CHD) following applications of single lifestyle interventions, respectively. A possible explanation of the superior effect of D + E on cardiorespiratory fitness could be the greater weight loss induced by caloric restriction.

The present systematic review has several limitations. A major limitation is that no search for unpublished studies and any additional data sources and strategies (author contacts, trial registers) was performed. Another limitation is the fact that not all potential effect modifiers were accounted for. Often participants in different arms of trials did not receive equal numbers of contacts. It could be argued that contact time rather than the specific elements of the intervention affected participant’s weight and cardiovascular risk factor outcomes. Heterogeneity could be observed for some outcome parameters, probably introduced by differences between trials, including different D + E regimens. Publication bias cannot be excluded to affect the results on any meta-analysis; however, formal statistical testing did not suggest publication bias for the current analysis. Had there been evidence of publication bias and if more studies were available, regression techniques could be used to adjust for this [66].

Although many studies included in our analysis had a substantial dropout rate (see Table 1), intention-to-treat analyses were generally not conducted. However, dropouts were generally similar for all intervention groups. Taken together, 2/3 of the included trials were judged as being at high risk of bias. Therefore, the results should be interpreted in a conservative manner. Strengths of this research include the application of the network meta-analysis, as well as the fact that there was no evidence of inconsistency for most outcomes, and an overall sample size of 3,521 participants.

## Conclusions

The present network meta-analysis provides moderate-quality evidence that D + E induces moderate long-term weight loss and reduces blood lipids and blood pressure when compared to E or D as single interventional measures, respectively. In addition, the evidence suggests moderate superiority of D over E regarding anthropometric outcome parameters. The current findings seem to be clinically relevant for public health, in particular for encouraging a combination of diet and exercise for primary prevention of overweight and obesity. Future trials should investigate the long-term effects of different training modalities (aerobic, resistance or combined training) in combination with dietary interventions for the prevention and treatment of overweight and obesity.

## Competing interests

The authors declare that they have no competing interests.

## Authors’ contributions

LS extracted the data. LS, SD and GH conducted the data analysis, interpreted the results and drafted and finalized the manuscript. All authors read and approved the final manuscript.

## Supplementary Material

Additional file 1 **Full search strategy: PUBMED. Table S1**: sensitivity analysis (low risk of bias). **Table S2**: sensitivity analysis (study length: ≥24 months). **Table S3**: sensitivity analysis (study length, <24 months). **Table S4**: sensitivity analysis (obese participants). **Table S5**: sensitivity analysis (participants ≥50 years of age). **Table S6**: sensitivity analyses (direct pairwise and network meta-analysis, fixed effects models). **Table S7**: model fit statistics for the random and fixed effects network meta-analysis models. **Table S8**: estimates of the effects of diet + exercise vs. exercise from direct and indirect evidence in node split models. **Figure S1**: network graph. **Figure S2**: funnel plot for body weight (diet + exercise vs. diet). **Figure S3**: funnel plot for body weight (diet + exercise vs. exercise). **Figure S4**: funnel plot for body weight (diet vs. exercise). Click here for file
