# Supplementary material for: Impact of long-term lifestyle programmes on weight loss and cardiovascular risk factors in overweight/obese participants: a systematic review and network meta-analysis
Source: Syst Rev. 2014 Oct 30;3:130. doi: 10.1186/2046-4053-3-130 (PMC4227972; doi:10.1186/2046-4053-3-130)
Supplement: Additional file 1 — Full search strategy: PUBMED. Table S1: sensitivity analysis (low risk of bias). Table S2: sensitivity analysis (study length: ≥24 months). Table S3: sensitivity analysis (study length, <24 months). Table S4: sensitivity analysis (obese participants). Table S5: sensitivity analysis (participants ≥50 years of age). Table S6: sensitivity analyses (direct pairwise and network meta-analysis, fixed effects models). Table S7: model fit statistics for the random and fixed effects network meta-analysis models. Table S8: estimates of the effects of diet + exercise vs. exercise from direct and indirect evidence in node split models. Figure S1: network graph. Figure S2: funnel plot for body weight (diet + exercise vs. diet). Figure S3: funnel plot for body weight (diet + exercise vs. exercise). Figure S4: funnel plot for body weight (diet vs. exercise). [file 2046-4053-3-130-S1.docx]

**Supporting Information**

**Impact of long-term lifestyle programmes on weight loss and cardiovascular risk factors in overweight/obese particpants: a systematic review and network meta-analysis**

Lukas Schwingshackl; Sofia Dias; Georg Hoffmann

## Full search strategy

## PUBMED: 10612 results

## Cochrane Register of clinical trials: 7883 results

*("lifestyle" OR "exercise" OR "diet") AND ("body weight" OR "lipids") AND ("randomized controlled trial" OR "randomized" OR "clinical trials as topic" OR "placebo" OR "randomly" OR "trial") NOT ("animals" NOT "humans")*

| **Outcomes** | **No. of Studies** | **Sample Size** | **MD** | **95% CI** | **p-values** | **I^2^** |
| --- | --- | --- | --- | --- | --- | --- |
| **D+E vs. D** | | | | | | |
| BW (kg) | 6 | 1367 | -0.81 | [-1.67, 0.06] | 0.07 | 2% |
| FM (kg) | 3 | 529 | -0.78 | [-2.71, 1.16] | 0.43 | 55% |
| VO_2_ max (ml/kg/min) | 3 | 474 | 3.11 | [1.82, 4.40] | <0.00001 | 68% |
| **D+E vs. E** | | | | | | |
| BW (kg) | 5 | 860 | -5.47 | [-7.80, -3.14] | <0.00001 | 84% |
| FM (kg) | 3 | 523 | -5.52 | [-7.00, -4.04] | <0.00001 | 42% |
| VO_2_ max (ml/kg/min) | 3 | 468 | 2.13 | [1.49, 2.77] | <0.00001 | 38% |
| **D vs. E** | | | | | | |
| BW (kg) | 6 | 997 | -4.38 | [-6.40, -2.37] | <0.0001 | 81% |
| FM (kg) | 4 | 650 | -4.81 | [-5.87, -3.76] | <0.00001 | 0% |
| VO_2_ max (ml/kg/min) | 3 | 472 | -0.81 | [-1.88, 0.27] | 0.14 | 66% |

Table S1: Low risk of bias sensitivity analysis (random effect meta-analysis) of effect size (95% CIs) expressed as weighted MD for the effects of diet+exercise vs. diet, diet+exercise vs. exercise, and diet vs. exercise on anthropometric outcomes, and cardiorespiratory fitness.

BW, body weight; D, diet; D+E, diet and exercise; E, exercise; FM, fat mass; VO_2_ max, maximal oxygen uptake;

| **Outcomes** | **No. of Studies** | **Sample Size** | **MD** | **95% CI** | **p-values** | **I^2^** |
| --- | --- | --- | --- | --- | --- | --- |
| **D+E vs. D** | | | | | | |
| BW (kg) | 6 | 549 | -1.82 | [-2.96, -0.69] | 0.002 | 0% |
| WC (cm) | 3 | 196 | -1.28 | [-3.29, 0.73] | 0.21 | 0% |
| **D+E vs. E** | | | | | | |
| BW (kg) | 3 | 376 | -1.96 | [-3.31, -0.61] | 0.005 | 0% |
| **D vs. E** | | | | | | |
| BW (kg) | 3 | 377 | -0.44 | [-2.36, 1.49] | 0.46 | 46% |

Table S2: Pooled estimates (including trials ≥24 months) of effect size (95% confidence intervals) expressed as weighted MD for the effects of diet+exercise vs. diet, diet+exercise vs. exercise, and diet vs. exercise on anthropometric outcomes.

BW, body weight; D, diet; E, exercise; WC, waist circumference.

| **Outcomes** | **No. of Studies** | **Sample Size** | **MD *** | **95% CI** | **p-values** | **I^2^** |
| --- | --- | --- | --- | --- | --- | --- |
| **D+E vs. D** | | | | | | |
| BW (kg) | 11 | 1768 | -1.21 | [-1.91, -0.51] | 0.0007 | 0% |
| WC (cm) | 5 | 928 | -1.82 | [-2.99, -0.65] | 0.002 | 4% |
| FM (kg) | 7 | 864 | -1.45 | [-2.66, -0.25] | 0.02 | 63% |
| WHR (U) | 4 | 511 | -0.01 | [-0.02, -0.01] | <0.00001 | 0% |
| TC (mg/dl) | 7 | 1060 | -4.88 | [-10.24, 0.48] | 0.07 | 50% |
| LDL-C (mg/dl) | 6 | 1032 | -3.93 | [-8.22, 0.36] | 0.07 | 41% |
| HDL-C (mg/dl) | 7 | 1060 | 1.85 | [0.28, 3.43] | 0.02 | 57% |
| TG (mg/dl) | 7 | 1060 | -11.47 | [-19.43, -3.50] | 0.005 | 0% |
| DBP (mmHg) | 6 | 1032 | -1.11 | [-2.20, -0.01] | 0.05 | 31% |
| SBP (mmHg) | 6 | 1032 | -0.08 | [-1.32, 1.15] | 0.89 | 0% |
| VO_2_ max (ml/kg/min) | 4 | 626 | 3.95 | [2.54, 5.35] | <0.00001 | 77% |
| **D+E vs. E** | | | | | | |
| BW (kg) | 6 | 974 | -4.57 | [-5.31, -3.83] | <0.00001 | 80% |
| WC (cm) | 3 | 409 | -3.00 | [-5.81, -0.20] | 0.04 | 69% |
| FM (kg) | 5 | 485 | -4.47 | [-6.29, -2.66] | <0.00001 | 68% |
| WHR (U) | 3 | 357 | -0.01 | [-0.02, 0.00] | 0.09 | 32% |
| TC (mg/dl) | 3 | 357 | -11.68 | [-16.59, -6.77] | <0.00001 | 0% |
| LDL-C (mg/dl) | 3 | 357 | -11.22 | [-15.58, -6.87] | <0.00001 | 0% |
| HDL-C (mg/dl) | 3 | 357 | -0.22 | [-3.11, 2.68] | 0.88 | 80% |
| TG (mg/dl) | 3 | 357 | -7.90 | [-22.67, 6.86] | 0.29 | 31% |
| DBP (mmHg) | 3 | 357 | -2.05 | [-3.44, -0.65] | 0.004 | 0% |
| SBP (mmHg) | 3 | 357 | -2.67 | [-4.42, -0.91] | 0.003 | 0% |
| VO_2_ max (ml/kg/min) | 3 | 468 | 2.18 | [1.33, 3.03] | <0.00001 | 38% |
| **D vs. E** | | | | | | |
| BW (kg) | 10 | 1261 | -3.30 | [-3.94, -2.66] | <0.00001 | 68% |
| WC (cm) | 4 | 539 | -1.75 | [-4.12, 0.62] | 0.15 | 71% |
| FM (kg) | 9 | 964 | -2.20 | [-3.75, -0.66] | 0.005 | 82% |
| WHR (U) | 3 | 348 | 0.00 | [-0.01, 0.01] | 0.99 | 27% |
| TC (mg/dl) | 6 | 599 | -2.74 | [-6.52, 1.04] | 0.16 | 0% |
| LDL-C (mg/dl) | 6 | 599 | -2.34 | [-5.63, 0.96] | 0.16 | 0% |
| HDL-C (mg/dl) | 6 | 599 | -0.94 | [-1.90, 0.01] | 0.05 | 0% |
| TG (mg/dl) | 6 | 599 | -3.71 | [-12.17, 4.74] | 0.39 | 0% |
| DBP (mmHg) | 5 | 507 | -1.69 | [-3.53, 0.14] | 0.07 | 40% |
| SBP (mmHg) | 5 | 512 | -2.28 | [-4.65, 0.08] | 0.06 | 36% |
| VO_2_ max (ml/kg/min) | 4 | 510 | -1.48 | [-2.90, -0.06] | 0.04 | 80% |

Table S3: Pooled estimates (including trials <24 months) of effect size (95% confidence intervals) expressed as weighted MD for the effects of diet+exercise vs. diet, diet+exercise vs. exercise, and diet vs. exercise on anthropometric outcomes blood lipids, blood pressure, and cardiorespiratory fitness.

BW, body weight; CI, confidence intervals; D, diet; DBP, diastolic blood pressure; D+E, diet and exercise; E, exercise; FM, fat mass; HDL-C, high density lipoprotein cholesterol; LDL-C, low-density lipoprotein cholesterol; SBP, systolic blood pressure; TC, total cholesterol; TG, triacyglycerols; WC, waist circumference; WHR, waist-to-hip ratio; VO_2_ max, maximal oxygen uptake;

Table S4: Pooled estimates (including obese participants) of effect size (95% confidence intervals) expressed as weighted MD for the effects of diet+exercise vs. diet, diet+exercise vs. exercise, and diet vs. exercise on anthropometric outcomes blood lipids, blood pressure, and cardiorespiratory fitness.

| **Outcomes** | **No. of Studies** | **Sample Size** | **MD*** | **95% CI** | **p-values** | **Inconsistency I^2^** |
| --- | --- | --- | --- | --- | --- | --- |
| **D+E vs. D** | | | | | | |
| BW (kg) | 12 | 1580 | -1.18 | [-2.17, -0.19] | 0.02 | 0% |
| WC (cm) | 5 | 725 | -1.19 | [-2.73, 0.35] | 0.13 | 0% |
| FM (kg) | 6 | 532 | -1.79 | [-3.50, -0.07] | 0.04 | 51% |
| WHR (U) | 3 | 191 | -0.01 | [-0.02, 0.01] | 0.40 | 0% |
| TC (mg/dl) | 5 | 672 | -3.86 | [-14.22, 6.50] | 0.47 | 61% |
| LDL-C (mg/dl) | 4 | 644 | 0.08 | [-7.99, 8.14] | 0.99 | 53% |
| HDL-C (mg/dl) | 5 | 672 | 0.83 | [-0.80, 2.46] | 0.32 | 0% |
| TG (mg/dl) | 5 | 672 | -12.80 | [-28.12, 2.52] | 0.10 | 0% |
| DBP (mmHg) | 4 | 644 | -1.32 | [-4.10, 1.46] | 0.35 | 44% |
| SBP (mmHg) | 4 | 644 | -1.01 | [-4.69, 2.67] | 0.59 | 33% |
| VO_2_ max (ml/kg/min) | 4 | 626 | 3.95 | [2.54, 5.35] | 0.19 | 51% |
| **D+E vs. E** | | | | | | |
| BW (kg) | 6 | 822 | -3.74 | [-6.38, -1.09] | 0.006 | 82% |
| FM (kg) | 3 | 352 | -3.92 | [-5.96, -1.88] | 0.0002 | 67% |
| **D vs. E** | | | | | | |
| BW (kg) | 8 | 992 | -2.65 | [-5.28, -0.03] | 0.05 | 83% |
| FM (kg) | 5 | 509 | -2.49 | [-4.78, -0.20] | 0.03 | 79% |
| TC (mg/dl) | 4 | 290 | -2.21 | [-12.15, 7.73] | 0.20 | 55% |
| LDL-C (mg/dl) | 4 | 290 | -2.12 | [-10.34, 6.09] | 0.33 | 66% |
| HDL-C (mg/dl) | 3 | 290 | -1.26 | [-3.11, 0.59] | 0.18 | 0% |
| TG (mg/dl) | 3 | 290 | -9.16 | [-20.88, 2.56] | 0.13 | 0% |
| DBP (mmHg) | 4 | 290 | -3.05 | [-6.38, 0.27] | 0.07 | 47% |
| SBP (mmHg) | 4 | 292 | -2.26 | [-6.89, 2.37] | 0.34 | 45% |
| VO_2_ max (ml/kg/min) | 3 | 156 | -0.91 | [-3.70, 1.88] | 0.52 | 89% |

BW, body weight; CI, confidence intervals; D, diet; DBP, diastolic blood pressure; D+E, diet and exercise; E, exercise; FM, fat mass; HDL-C, high density lipoprotein cholesterol; LDL-C, low-density lipoprotein cholesterol; SBP, systolic blood pressure; TC, total cholesterol; TG, triacyglycerols; VO_2_ max, maximal oxygen uptake; WC, waist circumference; WHR, waist-to-hip ratio;

| **Outcomes** | **No. of Studies** | **Sample Size** | **MD*** | **95% CI** | **p-values** | **I^2^** |
| --- | --- | --- | --- | --- | --- | --- |
| **D+E vs. D** | | | | | | |
| BW (kg) | 8 | 1422 | -0.83 | [-1.69, 0.03] | 0.06 | 0% |
| WC (cm) | 3 | 755 | -2.11 | [-5.38, 1.15] | 0.11 | 58% |
| FM (kg) | 4 | 556 | -1.46 | [-3.85, 0.94] | 0.23 | 66% |
| TC (mg/dl) | 4 | 735 | -6.10 | [-14.52, 2.31] | 0.16 | 64% |
| LDL-C (mg/dl) | 3 | 707 | -4.54 | [-11.91, 2.84] | 0.23 | 63% |
| HDL-C (mg/dl) | 4 | 735 | 0.27 | [-1.35, 1.90] | 0.74 | 27% |
| TG (mg/dl) | 4 | 735 | -6.35 | [-18.84, 6.13] | 0.32 | 0% |
| DBP (mmHg) | 3 | 707 | -1.35 | [-3.12, 0.41] | 0.13 | 54% |
| SBP (mmHg) | 3 | 707 | -0.31 | [-2.47, 1.84] | 0.78 | 33% |
| VO_2_ max (ml/kg/min) | 3 | 474 | 3.11 | [1.82, 4.40] | <0.00001 | 68% |
| **D+E vs. E** | | | | | | |
| BW (kg) | 5 | 860 | -5.47 | [-7.80, -3.14] | <0.00001 | 84% |
| FM (kg) | 3 | 523 | -5.52 | [-7.00, -4.04] | <0.00001 | 42% |
| VO_2_ max (ml/kg/min) | 3 | 468 | 2.18 | [1.33, 3.03] | <0.00001 | 38% |
| **D vs. E** | | | | | | |
| BW (kg) | 7 | 1035 | -4.15 | [-6.07, -2.22] | <0.00001 | 78% |
| FM (kg) | 5 | 688 | -3.97 | [-5.66, -2.29] | <0.00001 | 61% |
| TC (mg/dl) | 3 | 347 | -4.38 | [-9.30, 0.55] | 0.08 | 0% |
| LDL-C (mg/dl) | 3 | 347 | -3.30 | [-7.60, 1.00] | 0.13 | 0% |
| HDL-C (mg/dl) | 3 | 347 | -1.83 | [-3.15, -0.51] | 0.006 | 0% |
| TG (mg/dl) | 3 | 347 | -2.98 | [-13.30, 7.34] |  |  |
| DBP (mmHg) | 3 | 344 | -1.36 | [-3.54, 0.82] | 0.22 | 42% |
| SBP (mmHg) | 3 | 349 | -1.91 | [-5.38, 1.56] | 0.28 | 57% |
| VO_2_ max (ml/kg/min) | 4 | 510 | -1.48 | [-2.90, -0.06] | 0.04 | 80% |

Table S5: Pooled estimates (including participants ≥50 years of age) of effect size (95% confidence intervals) expressed as weighted MD for the effects of diet+exercise vs. diet, diet+exercise vs. exercise, and diet vs. exercise on anthropometric outcomes, blood lipids, blood pressure, and cardiorespiratory fitness.

BW, body weight; CI, confidence intervals; D, diet; DBP, diastolic blood pressure; D+E, diet and exercise; E, exercise; FM, fat mass; HDL-C, high density lipoprotein cholesterol; LDL-C, low-density lipoprotein cholesterol; SBP, systolic blood pressure; TC, total cholesterol; TG, triacyglycerols; VO_2_ max, maximal oxygen uptake; WC, waist circumference; WHR, waist-to-hip ratio;

Table S6: Estimates (direct pairwise and network meta-analysis, fixed effects models) of effect size (95% confidence intervals/ 95% credible intervals)expressed as mean difference for the effects of diet+exercise vs. diet, diet+exercise vs. exercise, and diet vs. exercise on anthropometric outcomes, blood lipids, blood pressure, and cardiorespiratory fitness.

| **Outcomes** | **No. of Studies** | **Sample Size** | **MD** | **95% CI** | **MD** | **95% CrI** |
| --- | --- | --- | --- | --- | --- | --- |
| **D+E vs. D** | | | | | | |
| BW (kg) | 17 | 2317 | -1.38 | [-1.98, -0.79] | -1.28 | [-1.80, -0.61] |
| WC (cm) | 8 | 1124 | -1.68 | [-2.66, -0.70] | -1.58 | [-2.55, -0.62] |
| FM (kg) | 9 | 1012 | -0.74 | [-1.32, -0.17] | -0.67 | [-1.23, -0.12] |
| WHR (U) | 6 | 646 | -0.01 | [-0.02, -0.01] | -0.01 | [-0.02, -0.007] |
| TC (mg/dl) | 9 | 1175 | -1.73 | [-4.86, 1.41] | -2.10 | [-5.19, 0.98] |
| LDL-C (mg/dl) | 8 | 1147 | -0.98 | [-3.75, 1.79] | -1.48 | [-4.20, 1.23] |
| HDL-C (mg/dl) | 9 | 1175 | 1.72 | [0.87, 2.57] | 1.68 | [0.83, 2.51] |
| TG (mg/dl) | 9 | 1175 | -10.08 | [-17.38, -2.79] | -8.85 | [-15.88, -1.84] |
| DBP (mmHg) | 7 | 1099 | -1.02 | [-1.84, -0.20] | -0.97 | [-1.78, -0.15] |
| SBP (mmHg) | 7 | 1099 | -0.24 | [-1.45, 0.97] | -0.33 | [-1.53, 0.86] |
| VO_2_ max (ml/kg/min) | 6 | 810 | 2.72 | [2.20, 3.25] | 2.75 | [2.23, 3.28] |
| **D+E vs. E** | | | | | | |
| BW (kg) | 9 | 1350 | -3.97 | [-4.62, -3.32] | -4.01 | [-4.64, -3.39] |
| WC (cm) | 3 | 409 | -3.75 | [-5.05, -2.44] | -3.73 | [-4.90, -2.56] |
| FM (kg) | 5 | 690 | -2.16 | [-2.78, -1.55] | -2.50 | [-3.07, -1.92] |
| WHR (U) | 4 | 420 | -0.01 | [-0.02, -0.00] | -0.019 | [-0.026, -0.01] |
| TC (mg/dl) | 4 | 420 | -11.36 | [-15.93, -6.79] | -7.67 | [-11.57, -3.76] |
| LDL-C (mg/dl) | 4 | 420 | -10.20 | [-14.24, -6.16] | -6.12 | [-9.56, -2.70] |
| HDL-C (mg/dl) | 4 | 420 | 0.18 | [-0.94, 1.29] | 0.77 | [0.24, 1.77] |
| TG (mg/dl) | 4 | 420 | -7.87 | [-19.36, 3.63] | -12.20 | [-21.22, -3.23] |
| DBP (mmHg) | 4 | 420 | -2.06 | [-3.39, -0.72] | -1.93 | [-3.13, -0.73] |
| SBP (mmHg) | 4 | 420 | -2.84 | [-4.54, -1.13] | -2.64 | [-4.21, -1.07] |
| VO_2_ max (ml/kg/min) | 5 | 645 | 2.12 | [1.55, 2.69] | 2.11 | [1.56, 2.65] |
| **D vs. E** | | | | | | |
| BW (kg) | 13 | 1638 | -2.76 | [-3.33, -2.18] | -2.80 | [-3.35, -2.26] |
| WC (cm) | 4 | 539 | -2.82 | [-3.96, -1.68] | -2.14 | [-3.24, -1.06] |
| FM (kg) | 9 | 964 | -1.83 | [-2.42, -1.25] | -1.82 | [-2.40, -1.25] |
| WHR (U) | 4 | 414 | -0.00 | [-0.01, 0.01] | -0.006 | [-0.012, 0.00] |
| TC (mg/dl) | 7 | 665 | -4.12 | [-7.71, -0.53] | -5.56 | [-9.02, -2.09] |
| LDL-C (mg/dl) | 7 | 665 | -3.26 | [-6.43, -0.09] | -4.64 | [-7.67, -1.59] |
| HDL-C (mg/dl) | 7 | 665 | -0.96 | [-1.88, -0.04] | -0.91 | [-1.84, -0.01] |
| TG (mg/dl) | 7 | 665 | -3.80 | [-12.21, 4.62] | -3.36 | [-11.31, 4.54] |
| DBP (mmHg) | 6 | 573 | -1.01 | [-2.22, 0.20] | -0.96 | [-2.12, 0.18] |
| SBP (mmHg) | 6 | 578 | -2.10 | [-3.71, -0.48] | -2.31 | [-3.85, -0.77] |
| VO2 max (ml/kg/min) | 6 | 677 | -0.60 | [-1.13, -0.07] | -0.64 | [-1.14, -0.13] |

Table S7: Model fit statistics for the random and fixed effects NMA models: the DIC is obtained as the sum of resdev, the posterior mean of the residual deviance and pD, the effective number of parameters estimated in the model.

|  |  |  | RE |  |  | FE |  |
| --- | --- | --- | --- | --- | --- | --- | --- |
| Outcome | data points | resdev | pD | DIC | resdev | pD | DIC |
| BW | 59 | 56.1 | 45.9 | 102 | 130.4 | 26.0 | 156.4 |
| DBP | 30 | 32.0 | 18.3 | 50.3 | 35.5 | 14.0 | 49.5 |
| FM | 36 | 36.1 | 30.3 | 66.4 | 118.3 | 17.0 | 135.3 |
| HDL | 36 | 38.7 | 32.3 | 71.0 | 101.5 | 17.0 | 118.5 |
| LDC | 34 | 35.7 | 25.2 | 60.9 | 51.5 | 16.0 | 67.4 |
| SBP | 30 | 29.7 | 16.5 | 46.2 | 30.5 | 14.0 | 44.5 |
| TC | 36 | 40.7 | 25.7 | 66.4 | 53.5 | 17.0 | 70.4 |
| TG | 36 | 39.9 | 21.5 | 61.4 | 43.4 | 16.9 | 60.3 |
| VO2max | 24 | 24.5 | 22.0 | 46.5 | 87.1 | 11.0 | 98.1 |
| WC | 24 | 26.8 | 17.2 | 44.0 | 32.5 | 12.0 | 44.5 |
| WHR | 24 | 25.1 | 23.7 | 48.8 | 163.4 | 11.0 | 174.4 |

Yellow cells represent outcomes for which FE model is acceptable.

Table S8: Estimates of the effect of D+E vs E obtained from direct and indirect evidence in node-split models with 95%CrI and Bayesian p-value for the difference between direct and indirect evidence.

|  |  |  | Direct | | Indirect | |  |
| --- | --- | --- | --- | --- | --- | --- | --- |
| Outcome | t1 | t2 | median | 95% CrI | median | 95% CrI | p |
| BW | 1 | 3 | 3,63 | (2.07,5.21) | 6,51 | (3.37,9.59) | 0,10 |
| DBP | 1 | 3 | 2,19 | (0.52,4.06) | 4,78 | (0.65,9.40) | 0,25 |
| FM | 1 | 3 | 3,54 | (1.35,5.82) | 4,97 | (1.08,8.99) | 0,51 |
| HDL | 1 | 3 | 0,39 | (-4.03,4.92) | -1,37 | (-9.28,6.97) | 0,68 |
| LDC | 1 | 3 | 9,72 | (4.15,14.75) | -2,97 | (-13.07,6.22) | 0,02 |
| SBP | 1 | 3 | 2,83 | (0.20,5.47) | 1,83 | (-3.80,9.27) | 0,76 |
| TC | 1 | 3 | 10,85 | (5.10,16.16) | -0,41 | (-9.91,9.28) | 0,05 |
| TG | 1 | 3 | 11,31 | (-4.67,31.80) | 21,19 | (-3.89,50.04) | 0,48 |
| VO2max | 1 | 3 | -2,14 | (-3.10,-1.23) | -0,48 | (-4.30,3.29) | 0,39 |
| WC | 1 | 3 | 3,27 | (-0.32,5.97) | 4,98 | (-1.19,11.90) | 0,54 |
| WHR | - | - |  |  |  |  |  |

Diet + Exercise

Diet

Exercise

Figure S1: Network of treatments compared. A line connecting two treatments means that they have been compared in a trial. The triangle represents comparisons made in one or more 3-arm trials, straight lines represent comparisons made in one or more in 2-arm trials. For the actual number of studies in each comparisons see Table 2.

The network of evidence for WHR does not have any 2-arm trials comparing diet with exercise.


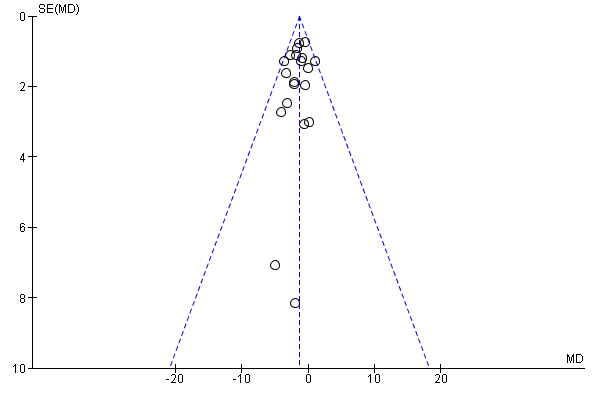


Figure S2: Funnel plot showing study precision against the MD effect estimate with 95% CIs for body weight (diet + exercise vs. diet). SE = Standard error


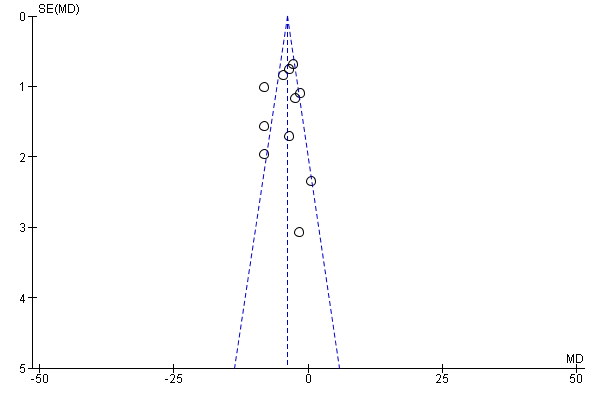
 Figure S3: Funnel plot showing study precision against the MD effect estimate with 95% CIs for body weight (diet + exercise vs. exercise). SE = Standard error


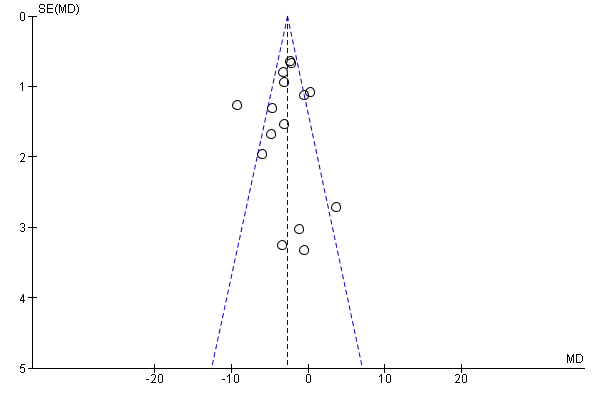


Figure S4: Funnel plot showing study precision against the MD effect estimate with 95% CIs for body weight (diet vs. exercise). SE = Standard error
